# Relationship between thyroid function and ICU mortality: a prospective observation study

**DOI:** 10.1186/cc11151

**Published:** 2012-01-19

**Authors:** Feilong Wang, Wenzhi Pan, Hairong Wang, Shuyun Wang, Shuming Pan, Junbo Ge

**Affiliations:** 1 Department of Emergency, Xinhua Hospital of Shanghai Jiaotong, No. 1665, Kongjiang Road, Shanghai, 200092, China; 2 Department of Cardiology, Shanghai Institute of Cardiovascular Diseases, Zhongshan Hospital of Fudan University, No. 180, Fenglin Road, Shanghai, 200032, China

## Abstract

**Introduction:**

Although nonthyroidal illness syndrome is considered to be associated with adverse outcome in ICU patients, the performance of thyroid hormone levels in predicting clinical outcome in ICU patients is unimpressive. This study was conducted to assess the prognostic value of the complete thyroid indicators (free triiodothyronine (FT3), total triiodothyronine; free thyroxine, total thyroxine, thyroid-stimulating hormone and reverse triiodothyronine) in unselected ICU patients.

**Methods:**

A total of 480 consecutive patients without known thyroid diseases were screened for eligibility and followed up during their ICU stay. We collected each patient's baseline characteristics, including the Acute Physiology and Chronic Health Evaluation II (APACHE II) score and thyroid hormone, N-terminal pro-brain natriuretic peptide (NT-proBNP) and C-reactive protein (CRP) levels. The primary outcome was ICU mortality. Potential predictors were analyzed for possible association with outcomes. We also evaluated the ability of thyroid hormones together with APACHE II score to predict ICU mortality by calculation of net reclassification improvement (NRI) and integrated discrimination improvement (IDI) indices.

**Results:**

Among the thyroid hormone indicators, FT3 had the greatest power to predict ICU mortality, as suggested by the largest area under the curve (AUC) of 0.762 ± 0.028. The AUC for FT3 level was less than that for APACHE II score (0.829 ± 0.022) but greater than that for NT-proBNP level (0.724 ± 0.030) or CRP level (0.689 ± 0.030). Multiple regression analysis revealed that FT3 level (standardized β = -0.600, *P *= 0.001), APACHE II score (standardized β = 0.912, *P *< 0.001), NT-proBNP level (standardized β = 0.459, *P *= 0.017) and CRP level (standardized β = 0.367, *P *= 0.030) could independently predict primary outcome. The addition of FT3 level to APACHE II score gave an NRI of 54.29% (*P *< 0.001) and an IDI of 36.54% (*P *< 0.001). The level of FT3 was significantly correlated with NT-proBNP levels (*r *= -0.344, *P *< 0.001) and CRP levels (*r *= -0.408, *P *< 0.001).

**Conclusion:**

In unselected ICU patients, FT3 was the most powerful and only independent predictor of ICU mortality among the complete indicators. The addition of FT3 level to the APACHE II score could significantly improve the ability to predict ICU mortality.

## Introduction

During critical illness, changes in circulating hormone levels are a common phenomenon [1]. These alterations are correlated with the severity of morbidity and the outcomes of patients in ICUs [2,3]. Thyroid hormones play a key role in the maintenance of body growth by modulating metabolism and the immune system. In the 20th century, researchers found that thyroid dysfunction is associated with the mortality of patients admitted to the ICU [4-6]. These alterations in thyroid hormone levels are referred to as "euthyroid sick syndrome" [7,8] or "nonthyroidal illness syndrome" (NTIS) [9,10], which is characterized by low serum levels of free and total triiodothyronine (T3) and high levels of reverse T3 (rT3) accompanied by normal or low levels of thyroxine (T4) and thyroid-stimulating hormone (TSH). Subsequent studies confirmed the association between NTIS and adverse outcomes in patients with sepsis [11,12], multiple trauma [13], acute respiratory distress syndrome [14], respiratory failure [15] and mechanical ventilation [16], as well as in unselected ICU patients [5,6,17-23]. However, the performance of thyroid hormones as predictors of adverse outcomes in general ICU patients has been unimpressive until now.

First, the results of previous studies were inconsistent. Researchers in some studies [16,17] demonstrated that free triiodothyronine (FT3) levels in nonsurvivors were significantly lower than those in survivors, whereas other researchers [18] showed that there was no association between FT3 levels and ICU patient outcomes. Conflicting results also were reported in terms of other indicators, such as total triiodothyronine (TT3) [17-20], free thyroxine (FT4) [17,18,20,23], total thyroxine (TT4) [5,6,17-19,22] and TSH [5,6,17-23].

Second, most of these studies [5,6,17-23] were rather small and evaluated the prognostic value of some but not all thyroid hormone indicators. Until now, none of the thyroid hormone indicators has been found to be the best predictor of ICU mortality.

Third, researchers in a few studies have detected the independent predictive ability of thyroid hormones [5,17,18,20,23] or have assessed the ability of thyroid hormones together with a scoring system to predict ICU mortality [5,19]. In our previous study, we showed that N-terminal pro-brain natriuretic peptide (NT-proBNP) and C-reactive protein (CRP) are independent predictors of ICU mortality [24].

Whether thyroid hormone indicators can predict ICU mortality independently of both predictors is unclear. These variables' performance in predicting ICU mortality has not yet been compared. We therefore undertook a prospective, observational study of a large population of unselected medical ICU patients to detect the independent predictors of ICU mortality on the basis of the complete panel of thyroid hormone levels (FT3, TT3, FT4, TT4, TSH, rT3 and T3/rT3) and to evaluate the ability of thyroid hormone level together with Acute Physiology and Chronic Health Evaluation II (APACHE II) score to predict ICU mortality.

## Materials and methods

### Participants

This prospective, observational study involved consecutive adult patients admitted to the ICU of Xin-Hua Hospital, which is affiliated with Shanghai Jiaotong University School of Medicine, between January 2009 and March 2010. The population in the present study was described in our pervious study [24]. We decided *a priori*, however, to exclude patients who met the following criteria: (1) age younger than 18 years; (2) history of any thyroid diseases, such as hyperthyroidism, hypothyroidism and thyroid tumors; (3) thyroid nodule found by physical examination when admitted to the ICU; (4) pregnancy within the previous 6 months; and (5) undergoing any hormonal therapy except insulin use or taking oral amiodarone. Patients who died or were discharged from the ICU within 4 hours of admission were also excluded, because data collection from these patients was difficult. The study was approved by Shanghai Jiaotong University Xin Hua Hospital Ethics Committee (XHEC2011-011) and was carried out in accordance with the Declaration of Helsinki. Because this was an observational study and all laboratory indices observed were commonly measured for all patients in our ICU department, the need for written informed consent was waived by the ethical review board.

### Measurements

TSH, TT3, FT3, TT4 and FT4 levels were measured using the ADVIA Centaur immunoassay system (Siemens Healthcare Diagnostics Inc, Tarrytown, NY, USA). rT3 level was measured using the Maglumi 1000 Analyzer chemiluminescence immunoassay system (SNIBE Co, Ltd, Guandong, China). The normal ranges of serum hormone concentrations in our laboratory are as follows: FT3, 3.5 to 6.5 pmol/L; TT3, 0.60 to 1.81 ng/ml; FT4, 11.5 to 22.7 pmol/L; TT4, 45 to 109 ng/ml; TSH, 0.35 to 5.50 mIU/L; and rT3, 0.16 to 0.95 ng/ml. Intra-assay coefficients of variation for FT3 range from 2.35% to 3.08%; for TT3, they range from 1.45% to 3.18%; for FT4, they range from 2.23% to 3.33%; for TT4, they range from 1.19% to 3.15%; for TSH, they range from 2.1% to 3.8%; and for rT3, the coefficient of variation is 4.52%. Serum creatinine and albumin levels were measured using the Hitachi 7600-120 analyzer (Hitachi, Tokyo, Japan). We calculated the estimated glomerular filtration rate (eGFR) by using the abbreviated Modification of Diet in Renal Disease study equation [25]. Serum CRP levels were measured using the QuikRead CRP test kit (Orion Diagnostica, Espoo, Finland). Intra-assay coefficients of variation ranged from 2% at 140 mg/L to 15% at 9 mg/L. The NT-proBNP level was determined using the Elecsys electrochemiluminescence assay (Cobas e 411 analyzer; Roche Diagnostics; Mannheim, Germany). The reported total coefficients of variation are 4.4% at mean concentrations 248.9 ng/L and 3.91% at 5,449 ng/L, respectively. Blood samples were obtained from all eligible patients when they were admitted to ICU for measurement of the indicators mentioned previously.

### Study outcome

At baseline, demographic and clinical characteristics, including the APACHE II score (which can range from 0 to 71, with higher scores indicating more severe illness), were collected, then the patients were followed up during their hospitalization. The primary outcome of this analysis was death in the ICU due to any cause.

### Statistical analysis

Continuous variables are presented as mean values ± SD or medians and ranges, and categorical variables are expressed as percentages. CRP and NT-proBNP values were logarithmically normalized and are presented as log(CRP) and log(NT-proBNP) for statistical calculations, respectively, because they were very skewed. Baseline characteristics between survivors and nonsurvivors were compared with an unpaired Student's *t*-test or the Mann-Whitney *U *test for continuous variables and a χ^2 ^test or Fisher's exact test for categorical variables. Receiver operating characteristic (ROC) curves were used to examine the performance of variables in predicting ICU mortality. The area under the curve (AUC, that is, C-index) was calculated from the ROC curve. A statistically derived value based on the Youden's index that maximized the sum of the sensitivity and specificity was used to define the optimal cutoff value [26]. Univariate logistic regression analyses were performed to examine the association between mortality and each of the predictors separately. We also conducted forward stepwise multivariate logistic regression analysis to determine the independent predictors of ICU mortality. Criteria of *P *< 0.05 for entry and *P *≥ 0.10 for removal were imposed in this procedure. Cox & Snell *R*^2 ^and Nagelkerke *R*^2 ^correlation coefficients were calculated to assess the goodness of fit of the models [27]. ORs for the continuous variables were described using standardized ORs, which were associated with a 1-SD change in the variable. The increased discriminative predictive value of the FT3 level in addition to the APACHE II score was examined by calculation of net reclassification improvement (NRI) and integrated discrimination improvement (IDI) indices as described by Pencina *et al*. [28]. NRI is the net increase vs the net decrease in risk categories among case patients minus that among control participants. It requires that there exist *a priori *meaningful risk categories (we used less than 10%, 10% to 30%, 30% to 50% and more than 50% for the risk of ICU death) [24,29]. IDI is the difference in Yates slopes between models, in which the Yates slope is the mean difference in predicted probabilities between case patients and control participants [29]. A two-sided *P *value less than 0.05 was considered statistically significant. All analyses were performed using SPSS version 13.0 software (SPSS, Inc, Chicago, IL, USA).

## Results

### Baseline characteristics

A total of 480 consecutive patients (59.79% male, mean age 71.71 ± 15.52 years) were eligible for enrollment in this study. The baseline clinical and laboratory characteristics of the patients are listed in Table 1. On the basis of the normal ranges given above, 23 (4.79%), 53 (11.04%), 261 (54.38%) and 48 (10.00%) patients had low T3, low T4, low FT3 and low FT4 levels, respectively, and 17 (3.54%) and 30 (6.25%) patients had high TSH and rT3 levels, respectively. The mean APACHE II score was 12.91 ± 6.67 points. The primary reasons for ICU admission were cardiovascular disease and pulmonary disease. A total of 91 patients (19.13%) died during their ICU stay. The levels of TT3, TT4, FT3, FT4, TSH and T3/rT3 were lower in nonsurvivors than in survivors (all *P *< 0.01) (Table 1), but there were no significant differences in rT3 levels between the groups (*P *= 0.401) (Table 1). Compared with survivors (see Table 1), nonsurvivors were older (76.32 ± 13.28 years vs 70.62 ± 15.82 years, *P *= 0.0001) and had higher APACHE II scores (19.49 ± 6.85 vs 11.38 ± 5.60, *P *< 0.0001), higher levels of NT-proBNP (*P *< 0.0001) and CRP (*P *< 0.0001) and lower levels of hemoglobin (*P *< 0.0001) and eGFR (*P *< 0.001).

**Table 1:** Baseline clinical and laboratory characteristics of study subjects^a^

Characteristics	All	Survivors	Nonsurvivors	*P *values
Number	480	388	92	
Males (%)	59.79	60.87	59.54	0.815
Age (years)	71.71 ± 15.52	70.62 ± 15.82	76.32 ± 13.28	0.0001
Principal diagnosis leading to ICU admission (%)				
Pulmonary disease	32.50	33.51	28.26	0.334
Cardiovascular disease	36.67	37.89	31.52	0.255
Neurologic disease	7.50	6.96	9.78	0.355
Digestive disease	5.0	5.41	3.26	0.395
Renal insufficiency	1.88	1.55	3.26	0.276
Poisoning	3.75	4.38	1.09	0.135
Infectious disease and/or sepsis	4.38	2.58	11.96	< 0.0001
Trauma	2.08	2.58	0.00	0.120
Other	6.25	5.15	10.87	0.042
Accompanying infection (%)	44.38	41.75	55.43	0.018
Hemoglobin (g/L)	117.35 ± 23.77	119.51 ± 22.81	108.25 ± 25.67	< 0.0001
eGFR (mL/minute/1.73 m^2^)	66.13 (4.18 to 314.43)	71.63 (4.18 to 314.43)	39.95(4.25 to 238.71)	< 0.0001
APACHE II score (points)	12.93 ± 6.67	11.38 ± 5.60	19.49 ± 6.85	< 0.0001
NT-proBNP (ng/ml)	2843.50 (20.95 to 35,000.00)	2201.00 (20.95 to 35,000.00)	8700.00 (142.40 to 35,000.0)	< 0.0001
CRP (mg/L)	33.50 (1.00 to 160.00)	23.00 (1.00 to 160.00)	96.50 (6.00 to 160.00)	< 0.0001
Albumin (g/L)	33.67 ± 4.98	34.41 ± 4.64	30.56 ± 5.18	< 0.0001
Thyroid function				
TT3 (nmol/L)	1.11 ± 0.33	1.16 ± 0.32	0.89 ± 0.30	< 0.0001
TT4 (nmol/L)	71.16 ± 21.81	73.92 ± 20.88	59.52 ± 21.92	< 0.0001
FT3 (pmol/L)	3.42 ± 0.63	3.53 ± 0.60	2.95 ± 0.57	< 0.0001
FT4 (pmol/L)	15.54 ± 3.40	15.80 ± 3.29	14.48 ± 3.66	0.0008
rT3 (nmol/L)	0.30 (0.01 to 4.44)	0.30 (0.01 to 1.51)	0.30 (0.02 to 4.44)	0.4010
TSH (IU/mL)	0.80 (0.04 to 23.07)	0.87 (0.04 to 23.87)	0.60 (0.05 to 12.73)	0.0022
T3/rT3	3.36 (0.61 to 138.00)	3.62 (0.33 to 138.00)	2.61 (0.06 to 26.00)	0.0009

^a^APACHE II score, Acute Physiology and Chronic Health Evaluation II score; eGFR, estimated glomerular filtration rate; CRP, C-reactive protein; FT3, free triiodothyronine; FT4, free thyroxine; NT-proBNP, N-terminal pro-brain natriuretic peptide; rT3, reverse triiodothyronine; TSH, thyroid-stimulating-hormone; TT3, total triiodothyronine; TT4, total thyroxine; T3/rT3, the ratio of rT3 to T3.

### Value of indicators in predicting ICU mortality

**Figure 1:**
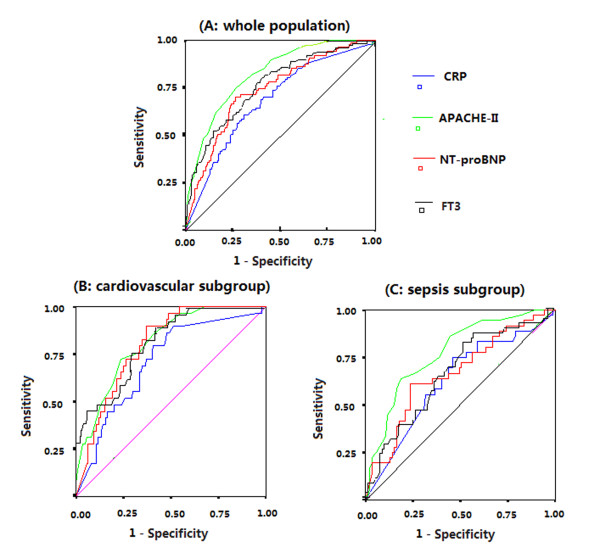
**Receiver operating characteristic curves for N-terminal pro-brain natriuretic peptide (NT-proBNP), C-reactive protein (CRP), free triiodothyronine (FT3) and Acute Physiology and Chronic Health Evaluation II (APACHE II) score in whole population (A), cardiovascular subgroup (B) and sepsis subgroup (C)**.

ROC curves were constructed to examine the performance of indicators as predictors of ICU mortality, then the AUC for each indicator was calculated. The AUC, optimal cutoff value, sensitivity and specificity of each indicator are given in Table 2. Among the thyroid hormone indicators, FT3 had the greatest power for predicting ICU mortality, as suggested by the largest AUC of 0.762 ± 0.028. The AUC for FT3 was less than that for APACHE II score (0.829 ± 0.022) but greater than that for NT-proBNP level (0.724 ± 0.030) or CRP level (0.689 ± 0.030), as shown in Figure 1A. We performed univariate logistic regression analyses to examine the association between the ICU mortality and each indicator and calculated the standardized coefficient (β) and OR for each variable (Table 3). FT3 had the greatest absolute value of standardized β (1.129) among the thyroid hormone indicators. The absolute value of standardized β for FT3 was also larger than that for NT-proBNP level (0.930) or CRP level (0.707), indicating that FT3 had greater power than NT-proBNP or CRP level for predicting ICU mortality.

**Table 2:** Performance of variables in predicting ICU mortality^a^

Variable	AUC ROC	*P *value	Cutoff value	Sensitivity (%)	Specificity (%)
TT3	0.722 ± 0.33	< 0.001	≤ 0.965	71.28	63.74
TT4	0.680 ± 0.031	< 0.001	≤ 7.04	57.18	72.53
FT3	0.762 ± 0.028	< 0.001	≤ 3.33	62.14	78.02
FT4	0.625 ± 0.035	< 0.001	≤ 13.63	74.93	49.46
rT3	0.529 ± 0.035	0.405			
TSH	0.603 ± 0.034	0.002	≤ 0.815	54.04	71.33
T3/rT3	0.617 ± 0.035	0.001	≤ 2.31	72.53	45.79
APACHE II score	0.829 ± 0.022	< 0.001	≥ 15	74.22	75.00
NT-proBNP	0.724 ± 0.030	< 0.001	≥ 5191	73.07	68.54
CRP	0.689 ± 0.030	< 0.001	≥ 66.5	69.66	61.11

^a^AUC ROC, area under the receiver operating characteristic curve; APACHE II score, Acute Physiology and Chronic Health Evaluation II score; CRP, C-reactive protein; FT3, free triiodothyronine; FT4, free thyroxine; NT-proBNP, N-terminal pro-brain natriuretic peptide; rT3, reverse triiodothyronine; TSH, thyroid-stimulating-hormone; TT3, total triiodothyronine; TT4, total thyroxine; T3/rT3, the ratio of rT3 to T3.

**Table 3:** Univariate odds ratios of variables for predicting ICU mortality^a^

Predictor	Standard β value	OR	95% CI	*P *value
TT3	-0.953	0.386	0.2889 to 0.516	< 0.001
TT4	-0.699	0.497	0.387 to 0.637	< 0.001
FT3	-1.129	0.323	0.239 to 0.436	< 0.001
FT4	-0.425	0.654	0.508 to 0.842	0.001
rT3	0.275	1.316	1.060 to 1.636	0.013
TSH	-0.263	0.769	0.537 to 1.100	0.151
T3/rT3	-0.765	0.465	0.230 to 0.940	0.031
Log(NT-proBNP)	0.930	2.530	1.876 to 3.425	< 0.001
Log(CRP)	0.707	2.028	1.563 to 2.632	< 0.001
APACHE II score	1.355	3.877	2.869 to 5.237	< 0.001

^a^APACHE II score, Acute Physiology and Chronic Health Evaluation II score; CRP, C-reactive protein; FT3, free triiodothyronine; FT4, free thyroxine; NT-proBNP, N-terminal pro-brain natriuretic peptide; rT3, reverse triiodothyronine; TSH, thyroid-stimulating-hormone; TT3, total triiodothyronine; TT4, total thyroxine; T3/rT3, the ratio of rT3 to T3. Standard β value was calculated using the semistandardization method (*X *standardization). Odds ratios are shown as standardized OR per 1 SD. Log variable is the logarithm of the variable.

### Independent predictive value of FT3

We conducted forward stepwise multivariate logistic regression analysis to determine the independent predictors of ICU mortality. The results are shown in Table 4. Among the thyroid hormone indicators, FT3 was the only independent predictor which entered the prediction models. When using the newest statistical analysis methods (NRI and IDI indices), we also found that addition of FT3 level to APACHE II score significantly improved the ability to predict outcomes. Addition of FT3 level to APACHE II score gave an NRI of 54.29% (*Z *value = 5.43, *P *< 0.001) and an IDI of 36.54% (*Z *value = 14.32, *P *< 0.001).

**Table 4:** Independent predictors of ICU mortality by multivariate logistic regression analysis in all patients (appending models summary)^a^

Predictor	Standard β value	OR	*P *value	-2 log-likelihood	Cox & Snell *R*^2^	Nagelkerke *R*^2^
Model I APACHE II score	1.355	3.877	0.000	315.784	0.186	0.301
Model II						
FT3	-0.741	0.477	0.000	295.291	0.225	0.364
APACHE II score	1.119	3.062	0.000			
Final model						
FT3	-0.600	0.549	0.001			
APACHE II score	0.912	2.490	0.000			
Log(NT-proBNP)	0.459	1.582	0.017	282.968	0.248	0.401
Log(CRP)	0.367	1.443	0.030			

^a^APACHE II score, Acute Physiology and Chronic Health Evaluation II score; eGFR, estimated glomerular filtration rate; FT3, free triiodothyronine; FT4, free thyroxine; NT-proBNP, N-terminal pro-brain natriuretic peptide; rT3 = reverse triiodothyronine; T3 = triiodothyronine; T4 = thyroxine; TSH = thyroid-stimulating hormone; TT3 = total triiodothyronine; TT4 = total thyroxine; T3/rT3 = ratio of rT3 to T3. Standard β value was calculated using the semistandardization method (*X *standardization). Odds ratios are shown as standardized OR per 1 SD. Log variable is the logarithm of the variable. Independent variables in the multivariate analysis included TT3, TT4, FT3, FT4, rT3, TSH, T3/rT3, Log(NT-proBNP), Log(CRP), APACHE II score and albumin (and age, eGFR and hemoglobin). Variables not listed in the table were removed from the stepwise analysis.

### Subgroup analysis

A total of 176 patients with cardiovascular disease as their principal diagnosis leading to ICU admission (59 patients with heart failure and 88 with acute coronary syndromes including acute myocardial infarction), and 130 patients with sepsis were included in the subgroup analysis. In the cardiovascular disease subgroup, the AUCs for the levels of FT3, NT-proBNP and CRP and the APACHE II score in the prediction of ICU mortality were 0.813 ± 0.038, 0.80 ± 0.036, 0.712 ± 0.050 and 0.816 ± 0.038, respectively (all *P *< 0.001) (Figure 1B). In the sepsis group, the AUCs for the levels of FT3, NT-proBNP and CRP and the APACHE II score to predict ICU mortality were 0.633 ± 0.055, 0.666 ± 0.054, 0.627 ± 0.056 and 0.755 ± 0.045, respectively (all *P *< 0.05) (Figure 1C). Patients were also divided into three subgroups for subgroup analysis based on their age: ages less than 60 (*n *= 105), 60 to 80 (*n *= 203) and more than 80 (*n *= 172) years. The AUCs, sensitivities, specificities and cutoff values for FT3 to predict ICU mortality were 0.795 ± 0.063 (*P *= 0.001), 75.00%, 72.43% and 3.30 pmol/L in the younger than age 60 years subgroup; 0.740 ± 0.046 (*P *< 0.001), 84.85%, 62.14% and 3.33 pmol/L in the age 60 to 80 years subgroup; and 0.762 ± 0.043 (*P *< 0.001), 53.19%, 89.69% and 2.92 pmol/L in older than age 80 years subgroup, respectively.

### Correlations of FT3 with other variables

The level of FT3 was negatively correlated with APACHE II score (*r *= -0.424, *P *< 0.001), age (*r *= -0.177, *P *< 0.001), NT-proBNP (*r *= -0.344, *P *< 0.001) and CRP (*r *= -0.408, *P *< 0.001), but positively associated with hemoglobin (*r *= 0.293, *P *< 0.001), albumin (*r *= 0.480, *P *< 0.001) and eGFR (r = 0.285, *P *< 0.001).

## Discussion

To the best of our knowledge, the present study is the largest clinical investigation of the prognostic value of thyroid hormones in ICU patients. In this study of 480 unselected ICU patients, we found that FT3 was the most powerful predictor of ICU mortality among the complete thyroid indicators (FT3, TT3, FT4, TT4, TSH, rT3 and T3/rT3) by calculation of AUC, standardized β and OR. FT3 had greater ability than NT-proBNP or CRP to predict primary outcome, as indicated by the larger AUC and standardized β value. Among the thyroid hormone indicators, FT3 was the only independent predictor of ICU mortality. Addition of FT3 to APACHE II score significantly improved the ability to predict primary outcomes, as demonstrated by the IDI and NRI indices.

In this study, we found that FT3 was the most powerful and only independent predictor of ICU mortality among the complete thyroid panel of indicators. Researchers in one previous study showed that there was no association between FT3 levels and adverse outcomes of ICU patients [18], and investigators in other studies showed that other indicators, such as TT3 [17,19], TT4 [5,18], FT4 [23] and TSH [19], were predictors of ICU mortality. However, the sample sizes of most of those studies were rather small, thus the conclusions drawn from them are less convincing. Why the results of the previous studies are different from those of our study can be attributed to two reasons. First, FT3 was not included in some of these previous studies [5,6,19]. The comparison of FT3 with other indicators in predicting outcomes was also not performed in these studies. The second reason may be attributed to the different populations included in the other studies. NTIS is a condition characterized by abnormal thyroid function tests encountered in patients with acute or chronic systemic illnesses. In the acute phase of critical illness, the alterations in thyroid hormones present as decreased T3 and increased T4 and rT3, as well as normal TSH [30]. In the chronic phase of critical illness, central hypothyroidism develops, and NTIS presents as decreased T3, decreased T4 and decreased TSH [30]. In the recovery phase of critical illness, the thyroidal axis begins with a rise in serum TSH, which is eventually followed by normalization in T4 concentration [30]. The conclusions drawn regarding the predictive value of thyroid hormones may be different in other studies because the patients included may have been in different phases of critical illness.

Among patients in different phases of critical illness, we found that the levels of T4, FT4 and TSH were increased, decreased or normal, but the T3 or FT3 level was generally reduced in patients with NTIS. Therefore, TT3 or FT3 level may be better than TSH and T4 level (or FT4 level) for predicting ICU outcomes. The TT3 or TT4 level can be affected by the concentration of thyroxine-binding globulin (TBG) or the binding ability of TBG, which may be affected by some health conditions, such as pregnancy and liver disease, and by a lot of commonly used drugs, including glucocorticoids, nonsteroidal anti-inflammatory drugs, furosemide and heparin. Conversely, FT3 and FT4 levels are not affected by these factors. Thus FT3 and FT4 levels may be better than TT3 and TT4 levels for predicting ICU outcomes. In our study, we also found that rT3 levels in survivors and nonsurvivors were similar and rT3 could not predict ICU mortality. An increase in rT3 level is the initial and most common abnormality observed in NTIS, owing to the inhibition of T4 conversion to rT3 by 5'-deiodinase. In patients with severe or chronic critical illness, however, central hypothyroidism develops, thus because T4 is decreased, rT3 levels do not further elevate. Therefore, rT3 levels may not correlate linearly with disease severity.

NT-proBNP and CRP were shown to be independent predictors of ICU mortality in our previous study [24], as well as in other studies. We found that FT3 had greater ability than NT-proBNP or CRP to predict primary outcomes in whole populations, as indicated by larger AUCs and greater standardized β values. In the present study, the predictive ability of FT3 level was independent of NT-proBNP and CRP levels as well as APACHE II scores. The addition of FT3 levels to APACHE II scores could significantly improve the ability to predict ICU mortality, as demonstrated by the IDI and NRI. In the subgroup of patients with cardiovascular disease, the AUC for FT3 (0.813 ± 0.038) was very close to that for NT-proBNP (0.801 ± 0.036) and APACHE II score (0.816 ± 0.038). In the sepsis subgroup, the AUC for FT3, NT-proBNP and CRP levels (0.633 ± 0.055, 0.666 ± 0.054 and 0.627 ± 0.056, respectively) were close but much smaller than the APACHE II score (0.755 ± 0.045). These results suggest that FT3 levels are more useful than NT-proBNP or CRP levels for predicting ICU mortality in unselected ICU patients and are not inferior to NT-proBNP or CRP levels in patients with cardiovascular disease or sepsis.

The pathophysiological mechanism underlying the association of lower FT3 levels with worse outcomes in ICU patients has yet to be fully defined. It is still unclear whether the alteration in thyroid hormone levels during critically illness is the adaptive physiological response to stress or the maladaptive response requiring treatment [30]. Researchers evaluating supplemental therapy for NTIS have also found contradictory results [31-33]. Inhibition of the enzyme 5'-deiodinase, which catalyzes the conversion of T4 to T3, has been considered a possible mechanism responsible for NTIS [30]. Several mechanisms can contribute to the inhibition of 5'-monodeiodination and thus to the low serum T3 concentrations in critically ill patients: cytokines (such as TNF, IFN-α, NF-κB and IL-6), some drugs (amiodarone and high doses of propranolol) and free (nonesterified) fatty acids. In the present study, we found that FT3 levels were most strongly correlated with CRP (*r *= -0.408, *P *< 0.001), indicating that inflammation or cytokines can significantly affect FT3 levels. Researchers in other studies have also found that inflammatory cytokines are associated with NTIS *in vitro *[34] and in clinical settings [35]. In our study, FT3 levels were also negatively correlated with NT-proBNP (*r *= -0.344, *P *< 0.001). This relationship indicated that low FT3 may be an indicator of poor cardiac function in ICU patients, because NT-proBNP levels have been shown to reflect hemodynamic stress and cardiac function in ICU patients [36]. Some studies have reported that patients with heart failure have low T3 serum concentrations, which correlates with cardiac function [37]. Others have found that patients with low T3 syndromes, but without overt cardiovascular disease, have an increased concentration of NT-proBNP, suggesting that low FT3 levels may be a contributing factor in the development of cardiac dysfunction [38]. Researchers in clinical studies have also found that low T3 syndromes have a negative prognostic effect in patients with heart failure [37]. Low FT3 levels have also been shown to be correlated with the frequent presence of, and adverse prognosis for, patients with coronary artery disease, even after adjusting for traditional coronary risk factors [39]. In short, low FT3 levels might represent an integrative measure of multiple harmful pathological processes occurring simultaneously in patients with critical illness, such as inflammation status and cardiac dysfunction, which are associated with adverse outcomes [24,40]. This hypothesis cannot completely explain the association of FT3 levels with adverse outcomes, however, because adjustment for previous biomarkers and clinical confounders in our study did not eliminate this association. Future studies are needed to explore further underlying mechanisms.

### Limitations

This study has several limitations. First, the inclusion of some patients with undetected thyroid disease before ICU admission may not be ruled out in the present study, even though we tested patients by palpation of the thyroid carefully when they were admitted to the ICU to exclude those with thyroid nodules. Second, although we excluded patients undergoing any replacement therapy except insulin use, as well as those taking oral amiodarone, it is clear that many other drugs (for example, propranolol, barbiturates, benzodiazepines, furosemide and dopamine) may have interfered with thyroid function, it is difficult to adjust for these potential confounders in clinical practice because so many drugs are involved and some increase and others decrease thyroid hormone levels. However, blood samples were obtained from patients at the time they were admitted to the ICU. Before we obtained blood samples, most of the patients had not been given these drugs. Additionally, many drugs cause abnormal thyroid function tests by affecting the concentration of TBG or the binding ability of TBG. Conversely, FT3 levels are not affected by these factors. So, the main conclusion of the study that FT3 was the most powerful and only independent predictor of ICU mortality among the complete thyroid indicators is relatively reliable.

## Conclusion

In this large-scale study of unselected ICU patients, we found that FT3 was the most powerful and only independent predictor of ICU mortality among the complete thyroid hormone indicators. FT3 had greater ability than NT-proBNP or CRP to predict primary outcomes. Addition of FT3 levels to APACHE II scores significantly improved the ability to predict ICU mortality, as demonstrated by IDI and NRI. The FT3 levels were negatively correlated with CRP and NT-proBNP levels.

## Key messages

• FT3 level was the most powerful and only independent predictor of ICU mortality among the complete thyroid indicators.

• Addition of the FT3 level to APACHE II score could significantly improve the ability to predict ICU mortality, as demonstrated by IDI and NRI indices.

• The level of FT3 was negatively correlated to APACHE II score, NT-proBNP and CRP.

## Abbreviations

AUC: area under the curve; APACHE II: Acute Physiology and Chronic Health Evaluation II; BNP: brain natriuretic peptide; CRP: C-reactive protein; eGFR: estimated glomerular filtration rate; FT3: free triiodothyronine; FT4: free thyroxine; IDI: integrated discrimination improvement; IFN: interferon; IL: interleukin; NF-κB: nuclear factor κB; NRI: net reclassification improvement; NTIS: nonthyroidal illness syndrome; ROC curve: receiver operating characteristic curve; rT3: reverse triiodothyronine; T3: triiodothyronine; T4: thyroxine; TNF: tumor necrosis factor; TSH: thyroid-stimulating hormone; TT3: total triiodothyronine; TT4: total thyroxine; T3/rT3: ratio of rT3 to T3.

## Competing interests

The authors declare that they have no competing interests.

## Authors' contributions

FW, HW and SP carried out data collection, contributed to the design of the study and helped to draft the manuscript. WP and JG participated in the design of the study, performed the statistical analysis and drafted the manuscript. SW participated in the data collection. All authors read and approved the final manuscript.
